# Impact of reduced margin pelvic radiotherapy on gastrointestinal toxicity and outcome in gynecological cancer

**DOI:** 10.1016/j.ctro.2023.100671

**Published:** 2023-08-28

**Authors:** Jie Lee, Jhen-Bin Lin, Chia-Sui Weng, Sue-Jar Chen, Tze-Chien Chen, Yu-Jen Chen

**Affiliations:** a Department of Radiation Oncology, MacKay Memorial Hospital, Taipei, Taiwan; b Department of Medicine, MacKay Medical College, New Taipei City, Taiwan; c Department of Radiation Oncology, Changhua Christian Hospital, Changhua, Taiwan; d Department of Obstetrics and Gynecology, MacKay Memorial Hospital, Taipei, Taiwan

**Keywords:** Reduced margin, Pelvic radiotherapy, Image-guided radiotherapy, Gynecological cancer, Gastrointestinal toxicity, Optimized outcome

## Abstract

**Purpose:**

To investigate the effect of reduced margin pelvic radiotherapy on gastrointestinal toxicity and outcomes in gynecological cancer.

**Materials and methods:**

This retrospective study analyzed data of 590 patients who underwent hysterectomy and adjuvant pelvic radiotherapy between 2010 and 2020 at two tertiary centers. The pelvic nodal region was delineated based on a reduced margin definition or the Radiation Therapy Oncology Group (RTOG) guidelines. All patients were treated with intensity-modulated radiotherapy with imaging guidance. Gastrointestinal toxicity was assessed using the Common Terminology Criteria for Adverse Events (CTCAE) and the Patient-Reported Outcome version (PRO-CTCAE).

**Results:**

Overall, 352 (59.7%) and 238 (40.3%) patients underwent RTOG and reduced margin pelvic radiotherapy, respectively. Median follow-up was 6.4 years (IQR: 3.7–9.6). Reduced margin pelvic radiotherapy significantly lowered the radiation dose to the small bowel. For CTCAE grade ≥ 2 or 3, acute gastrointestinal toxicity was lower in the reduced margin group than in the RTOG group (16.4% vs. 33.5%, *p* < 0.001; 2.9% vs. 8.5%, *p* < 0.001). The reduced margin group reported less severe acute gastrointestinal toxicity (PRO-CTCAE score ≥ 3) than the RTOG group (12.5% vs. 28.7%, *p* < 0.001). Late grade 3 gastrointestinal toxicity was lower in the reduced margin group than in the RTOG group (0.8% vs. 4.8%, *p* = 0.006). The 5-year pelvic recurrence-free survival and disease-free survival in the RTOG and reduced margin pelvic radiotherapy groups were 97.4% and 97.9% (*p* = 0.55) and 80.7% and 83.5% (*p* = 0.18), respectively.

**Conclusion:**

Reduced margin pelvic radiotherapy decreased acute and late gastrointestinal toxicity and achieved favorable outcomes.

## Highlights

• Reduced margin definition of pelvic radiotherapy was developed.
• Reduced margin pelvic radiotherapy decreases acute and late gastrointestinal toxicity.
• Measures included Patient-Reported Outcome for gastrointestinal toxicity.
• Reduced margin radiotherapy achieved favorable pelvic control and survival outcomes.

## 1 Introduction

Adjuvant pelvic radiotherapy for early-stage cervical cancer reduces the risk of pelvic recurrence in women with intermediate-risk features and improves survival in women with high-risk features [1]. It is recommended for endometrial cancer in women with intermediate- or high-risk features [2]. Although adjuvant pelvic radiotherapy benefits these patients, it is associated with acute and late gastrointestinal (GI) toxicity, which may impair their quality of life [3], [4]. With advances in radiation techniques, intensity-modulated radiotherapy (IMRT) can deliver conformal radiation to the pelvic nodal region while sparing organs at risk. Studies have also revealed that compared with conventional three-dimensional conformal radiotherapy, pelvic IMRT reduces the risk of GI toxicity [5], [6], [7], [8], [9]. To achieve the optimal therapeutic ratio for pelvic IMRT, a precise definition of the target volume based on the at-risk pelvic nodal region is essential.

The current pelvic nodal clinical target volume is mainly based on the Radiation Therapy Oncology Group (RTOG) guidelines [10], [11], [12], which suggest a uniform expansion of 7-mm margin surrounding the common, external, and internal iliac vessels. However, in patients who have undergone hysterectomy, the small bowel fills the pelvis and is frequently in direct contact with the vessels. The small bowel is a mobile organ that can move into RTOG-based target volume during radiotherapy, which may increase the radiation dose to the bowel. Therefore, a more precise pelvic nodal definition may help cover the at-risk nodal region while sparing the adjacent small bowel better. On the basis of pelvic nodal mapping, the pelvic nodes are more likely to be located in the lateral compartment of these vessels and less likely to be located in the medial compartment toward the pelvic cavity [12]. The great vessels in the pelvic cavity are also covered by the peritoneum, which has a thickness of approximately 200–600 μm in adults, suggesting that a smaller margin toward the pelvic cavity may be reasonable [13]. In addition, implementing image-guided radiotherapy techniques enables more accurate and precise delivery of radiation by using imaging before each fraction.

**Fig. 1:**
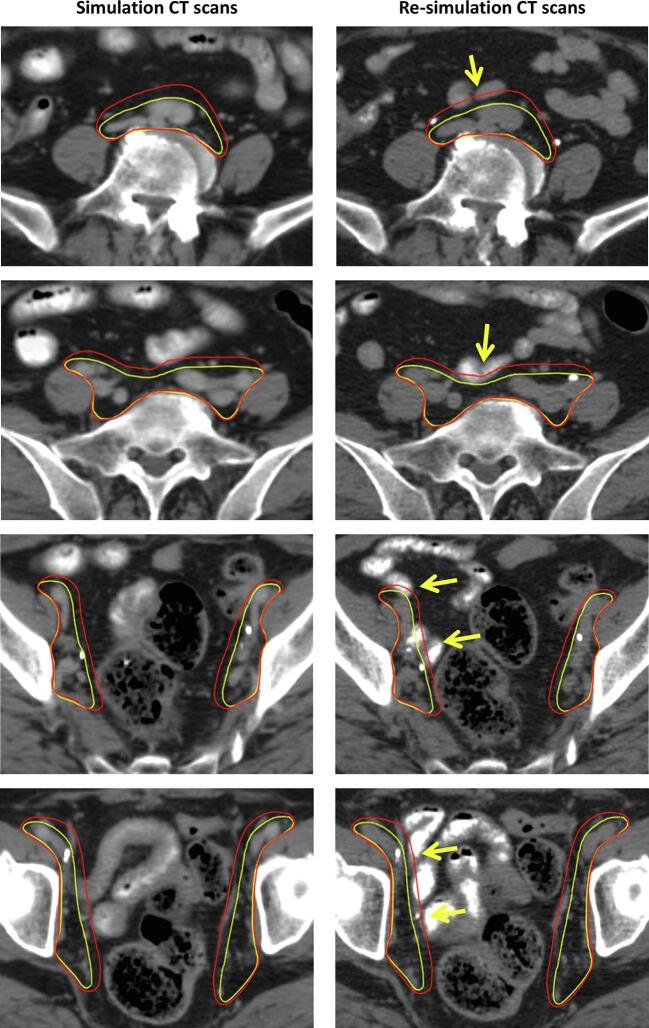
Illustrative clinical target volume based on the RTOG guideline (red) and reduced margin definition (yellow) on the simulation CT scans for radiotherapy (left). The clinical target volume of simulation CT scans was applied on the re-simulation CT scans during the course of radiotherapy (right). With regard to the bowel on the CT scans at two time points, the bowel was not included in the RTOG target volume on the initial simulation CT scans; however, the bowel was included the RTOG target volume during radiotherapy (yellow arrow), while with reduced margin definition, there was less inclusion of the bowel within the target volume. CT, computed tomography; RTOG, Radiation Therapy Oncology Group. (For interpretation of the references to colour in this figure legend, the reader is referred to the web version of this article.)

On the basis of these phenomena, we modified the definition of the pelvic nodal region to include a smaller margin from the vessels **(**Table 1 and Fig. 1**)**. We hypothesized that reduced margin pelvic radiotherapy with image guidance would decrease radiation to the bowel and GI toxicity without jeopardizing outcomes. Therefore, in this study, we aimed to evaluate the impact of image-guided reduced margin pelvic radiotherapy on toxicity and outcomes by comparing its results with those of RTOG-based pelvic radiotherapy.

**Table 1:** Reduced margin definition for pelvic radiotherapy.

**Nodal regions**	**Description**
Common iliac	Up to 3 mm anterior margin; 7 mm lateral margin around vessels extend posterior and lateral borders to psoas and vertebral body
External iliac	Up to 3 mm medial margin from vessels toward direction of pelvic cavity; 7–10 mm lateral margin from vessels.
Obturator	Join external and internal iliac regions with 10–15 mm wide strip along pelvic sidewall
Internal iliac	Up to 3 mm medial margin from vessels toward direction of pelvic cavity; 7 mm lateral margin from vessels.
Pre-sacral	10 mm strip over anterior to S1-S2 bodies.

## 2 Methods

### 2.1 Study population

This retrospective study was approved by the institutional review board of our institution. The data of 674 patients from two tertiary centers with International Federation of Gynecology and Obstetrics (FIGO) stage I–II cervical cancer (FIGO 2018) or FIGO stage I–III endometrial cancer (FIGO 2009) who underwent surgery and adjuvant pelvic radiotherapy between January 2010 and December 2020 were reviewed. For patients with cervical cancer, the surgery comprised radical hysterectomy and pelvic lymph node dissection. For patients with endometrial cancer, the surgery comprised total abdominal or laparoscopic hysterectomy, bilateral salpingo-oophorectomy, and lymph node dissection (pelvic alone or pelvic and *para*-aortic). In case of endometrial cancer with involvement of cervical stroma on preoperative imaging (computed tomography [CT] or magnetic resonance imaging), the surgery comprised radical hysterectomy and lymph node dissection (pelvic alone or pelvic and *para*-aortic).

Patients with prior malignancy (*n* = 10), prior abdominal or pelvic surgery (*n* = 12), missing GI toxicity data (*n* = 27), or missing follow-up data (*n* = 35) were excluded. Baseline patient, disease, and treatment characteristics, GI toxicity, and follow-up data were collected from institutional gynecological cancer databases.

### 2.2 Radiotherapy

The IMRT of all patients was planned using simulation CT in the supine position, and was delivered using 6- or 10-MV photons. A total dose of 45 or 50.4 Gy at 1.8 Gy per fraction was prescribed. The pelvic nodal clinical target volume encompassed the bilateral common iliac, external iliac, internal iliac, obturator, and presacral nodal vessels with uniform 7 mm margins based on the RTOG guidelines [10] or the reduced margin definition (Table 1 and Fig. 1). The principles for delineating the pelvic nodal clinical target volume were selected based on the preferences of the treating radiation oncologists. The vaginal clinical target volume was delineated based on the RTOG guidelines. The clinical target volume was expanded by 7 mm to generate the planning target volume based on institutional practice. The target planning constraints were as follows: (a) > 95% of the planning target volume received the prescription dose; (b) 0.03 cc of the planning target volume received < 110% of the prescription dose; and (c) < 1% of the planning target volume received < 93% of the prescription dose. The normal tissue planning constraints were as follows: (a) for the small bowel, <195 cc of the volume received ≥ 45 Gy, and < 40% of the volume received ≥ 30 Gy; (b) for the rectum and bladder, <50% of the volume received ≥ 40 Gy; and (c) the spinal cord received a maximum dose < 45 Gy. Daily online setup corrections were performed for all patients based on cone-beam CT. The dose–volume histogram data of the small bowel and rectum were derived from radiotherapy planning for each patient to compare the dose–volume of these organs, where Vx is the volume (mL) receiving a radiation dose of × (Gy).

### 2.3 Toxicity assessment and follow-up

Acute GI toxicities, including abdominal pain and diarrhea, were graded weekly by the treating physicians using the Common Terminology Criteria for Adverse Events (CTCAE), version 4.0. Abdominal pain and diarrhea were selected for assessment because they are the most common and clinically important GI toxicities experienced by patients during pelvic radiotherapy [3]. Since 2014, the Patient-Reported Outcome version of the CTCAE (PRO-CTCAE) questionnaire was also used to assess acute GI toxicity in our clinical practice. The questionnaire included questions on abdominal pain (severity, frequency, and interference with daily activities) and the frequency of diarrhea [14], [15], [16]. Patients scored toxicity on a 5-point Likert scale, with 0 indicating none, not at all, and never. The highest grade or score for each item during the 3–5 weeks of radiotherapy was analyzed because acute GI toxicities typically become symptomatic at 3 weeks and peak at 5 weeks. A grade or score of ≥ 3 was defined as severe GI toxicity. Late GI toxicity was defined as adverse events occurring at least 3 months after completion of radiotherapy. Late GI toxicity was graded by physicians using the CTCAE, version 4.0; a grade ≥ 3 was defined as severe late GI toxicity.

After completing adjuvant pelvic radiotherapy, patients were followed up with pelvic examinations every 3 months for 2 years and, subsequently, at intervals of 6–12 months. For patients with cervical cancer, Pap smears of cells from the vaginal cuff were undertaken every 6 months for 5 years and then annually. Post-treatment CT or magnetic resonance imaging scans were performed and evaluated at 3 and 6 months after treatment and then annually or when relapse was suspected.

### 2.4 Study endpoints

The primary endpoint was acute GI toxicity assessed using CTCAE or PRO-CTCAE. The secondary endpoints were the cumulative incidence of severe late GI toxicity, pelvic recurrence-free survival (PRFS), disease-free survival (DFS), and overall survival (OS). The time to severe late GI toxicity was measured from the date of surgery to the date of the event or the last follow-up. PRFS was defined as the time from the date of surgery to the date of pelvic recurrence, all-cause death, or last follow-up. OS was defined as the time from the date of surgery to the date of all-cause death or last follow-up, whereas DFS was defined as the time from the date of surgery to the date of disease recurrence, all-cause death, or last follow-up. Patterns of recurrence were defined as pelvic or distant, wherein pelvic recurrence was recurrence involving the pelvic lymph nodes and/or pelvic side walls, and distant recurrence included metastatic spread to organs, peritoneal carcinomatosis, and nodal recurrence in the mediastinal and supraclavicular regions.

### 2.5 Statistical analysis

Continuous data are presented as the medians and interquartile ranges (IQRs) or means ± standard deviations, as applicable, whereas categorical data are presented as numbers (%). Continuous variables were compared using the independent *t*-test or Mann–Whitney *U* test, and categorical variables were compared using the chi-square test. Logistic regression models were used to test the association between acute GI toxicity and other covariables. Cox proportional hazards models were used to estimate the hazard ratios and 95% confidence intervals. The variables included in the multivariable model were age, body mass index (BMI), Eastern Cooperative Oncology Group (ECOG) performance status, tumor site, hysterectomy type, surgical technique, chemotherapy, radiotherapy dose, and delineation. Survival curves were constructed using the Kaplan–Meier method with log-rank tests. We used SPSS (version 21.0; IBM Corp., Armonk, NY, USA) for statistical analyses. Statistical significance was set at *p* < 0.05.

## 3 Results

### 3.1 Patients

The final analysis included data from 590 patients, of whom 352 (59.7%) and 238 (40.3%) received RTOG and reduced margin pelvic radiotherapy, respectively.

The patient and tumor characteristics are shown in Table 2. Age, BMI, ECOG performance status, tumor stage, histology, types of surgery, chemotherapy use, and radiotherapy dose did not significantly differ between the RTOG and reduced margin pelvic radiotherapy groups. Pelvic nodal delineation with reduced margin achieved significantly lower dose–volume parameters of the small bowel and rectum than did RTOG pelvic nodal delineation, except that the V15 of the rectum was similar between the groups. Patients from both groups completed the course of pelvic radiotherapy within a similar duration. The median follow-up duration was 6.4 years (IQR: 3.7–9.6) in the overall cohort and was similar between the groups (*p* = 0.47) (Table 2).

**Table 2:** Demographic and clinical characteristics.

**Characteristics**	**RTOG** **(*n* = 352)**	**Reduced margin (*n* = 238)**	***p* value**
**Age (years), median (IQR)**	56 (49–62)	57 (51–63)	0.14
**BMI (kg/m^2^)**	22.7 ± 2.7	23.0 ± 2.7	0.29
**ECOG**			0.57
0	306 (86.9)	203 (85.3)	
1	46 (13.1)	35 (14.7)	
**Tumor stage**			0.95
Endometrium, FIGO (2009) stage I	88 (25.0)	62 (26.1)	
Endometrium, FIGO (2009) stage II	38 (10.8)	26 (10.9)	
Endometrium, FIGO (2009) stage III	124 (35.2)	76 (31.9)	
Cervix, FIGO (2018) stage I	75 (21.3)	55 (23.1)	
Cervix, FIGO (2018) stage II	27 (7.7)	19 (8.0)	
**Histology (endometrial cancer)**	*n* = 250	*n* = 164	0.91
Endometrioid, grade 1	44 (17.6)	30 (18.3)	
Endometrioid, grade 2	96 (38.4)	63 (38.4)	
Endometrioid, grade 3	77 (30.8)	49 (29.9)	
Serous	15 (6.0)	10 (6.1)	
Clear cell	8 (3.2)	8 (4.9)	
Other*	10 (4.0)	4 (2.4)	
**Histology (cervical cancer)**	*n* = 102	*n* = 74	0.89
Squamous cell carcinoma	74 (72.5)	53 (71.6)	
Adenocarcinoma	28 (27.5)	21 (28.4)	
**Types of surgery**			0.51
TAH/BSO + LND	104 (29.5)	60 (25.2)	
TLH/BSO + LND	119 (33.8)	85 (35.7)	
RH + LND	129 (36.6)	93 (39.1)	
**Chemotherapy**			0.68
No	204 (58.0)	142 (59.7)	
Yes	148 (42.0)	96 (40.3)	
**RT dose**			0.82
45 Gy	275 (78.1)	184 (77.3)	
50.4 Gy	77 (21.9)	54 (22.7)	
**Dose–volume parameters of small bowel**			
V15 (mL)	1116.6 ± 245.4	977.5 ± 188.1	<0.001
V30 (mL)	601.9 ± 133.0	526.2 ± 110.8	<0.001
V45 (mL)	169.0 ± 44.1	117.6 ± 36.9	<0.001
**Dose–volume parameters of rectum**			
V15 (mL)	74.1 ± 19.9	71.8 ± 16.6	0.15
V30 (mL)	56.6 ± 16.1	49.5 ± 12.5	<0.001
V45 (mL)	32.9 ± 11.0	24.2 ± 7.3	<0.001
**RT duration (day), median (IQR)**	37 (36–39)	37 (36–39)	0.89
**Follow-up (years), median (IQR)**	6.4 (3.5–9.6)	6.5 (3.8–9.6)	0.47

Abbreviations: BMI, body mass index; BSO, bilateral salpingo-oophorectomy; ECOG, Eastern Cooperative Oncology Group; FIGO, International Federation of Gynecology and Obstetrics; IQR, interquartile range; LND, lymph node dissection; RH, radical hysterectomy; RT, radiotherapy; RTOG, Radiation Therapy Oncology Group; TAH, total abdominal hysterectomy; TLH, total laparoscopic hysterectomy.

Data are presented as mean ± standard deviation or *n* (%).

* Other histology includes mucinous adenocarcinoma or undifferentiated carcinoma.

### 3.2 GI toxicity

Acute and late GI toxicities according to the radiotherapy group are shown in Table 3. No grade 4 or 5 toxicities were reported. The incidence of physician-reported CTCAE grade ≥ 2 or grade 3 acute GI toxicity, diarrhea, and abdominal pain was significantly lower in the reduced margin pelvic radiotherapy group than in the RTOG pelvic radiotherapy group.

**Table 3:** Comparison of acute and late toxicities by groups.

	**RTOG** **(*n* = 352)**	**Reduced margin (*n* = 238)**	***p* value**
**CTCAE acute GI toxicity**			
Any grade ≥ 2 toxicity	118 (33.5)	39 (16.4)	<0.001
Grade ≥ 2 diarrhea	90 (25.6)	24 (10.1)	<0.001
Grade ≥ 2 abdominal pain	72 (20.5)	22 (9.2)	<0.001
Any grade 3 toxicity	30 (8.5)	7 (2.9)	0.006
Grade 3 diarrhea	27 (7.7)	6 (2.5)	0.008
Grade 3 abdominal pain	21 (6.0)	5 (2.1)	0.03
PRO-CTCAE acute GI toxicity*	*n* = 178	*n* = 120	
Any score 3 toxicity	51 (28.7)	15 (12.5)	<0.001
Score 3 diarrhea	43 (24.2)	13 (10.8)	0.004
Score 3 abdominal pain, severity	24 (13.5)	6 (5.0)	0.02
Score 3 abdominal pain, frequency	22 (12.4)	5 (4.2)	0.02
Score 3 abdominal pain, interference	19 (10.7)	5 (4.2)	0.04
**Late GI toxicity**			
Grade ≥ 3 toxicity	17 (4.8)	2 (0.8)	0.007

Abbreviations: CTCAE, Common Terminology Criteria for Adverse Events; GI, gastrointestinal; PRO-CTCAE, Patient-Reported Outcome version; RTOG, Radiation Therapy Oncology Group.

Data are presented as *n* (%).

* Analysis of PRO-CTCAE in patients who received pelvic radiotherapy between 2014 and 2020.

Between 2014 and 2020, 221 and 153 patients received RTOG and reduced margin pelvic radiotherapy, respectively; among them, 178 (80.5%) and 120 (78.4%) patients, respectively, reported PRO-CTCAE GI toxicity. Patients who received reduced margin pelvic radiotherapy reported less severe acute GI toxicities than did those who received RTOG pelvic radiotherapy (12.5% vs. 28.7%, *p* < 0.001). Comparing the items of PRO-CTCAE GI toxicities between the groups, patients who received reduced margin pelvic radiotherapy reported significantly less severe diarrhea (frequency) and abdominal pain (severity, frequency, and interference) than did those who received RTOG pelvic radiotherapy. The results of the multivariable logistic regression analysis revealed that reduced margin pelvic radiotherapy was independently associated with lesser cases of CTCAE grade ≥ 2 and grade 3 acute GI toxicity, any PRO-CTCAE score 3 acute GI toxicity, and PRO-CTCAE score 3 diarrhea (Tables S1–S2).

**Fig. 2:**
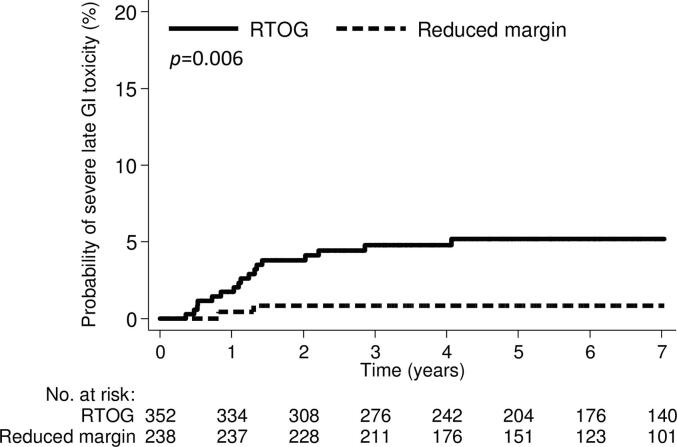
Cumulative incidence of severe (grade ≥ 3) late gastrointestinal toxicity.

Nineteen (3.2%) patients experienced severe late GI toxicity. All 19 patients underwent surgical intervention for severe late GI toxicities including enterocolitis, bowel obstruction, or perforation. Seventeen (4.8%) and two patients (0.8%) in the RTOG and reduced margin pelvic radiotherapy groups, respectively, experienced severe late GI toxicities (*p* = 0.007). The 3-year severe late GI toxicity rates for RTOG and reduced margin pelvic radiotherapy were 4.8% and 0.8%, respectively (*p* = 0.006, Fig. 2).

### 3.3 Treatment outcomes

There were 71 (20.2%) and 39 (16.4%) recurrent events, and 54 (15.3%) and 30 (12.6%) deaths in the RTOG and reduced margin groups, respectively. Distant recurrence was the predominant site of failure in both groups (19.9% vs. 16.0%, *p* = 0.23). Pelvic recurrence occurred in 10 (2.8%) and 5 (2.1%) patients in the RTOG and reduced margin groups, respectively (*p* = 0.58). In both groups, most sites of pelvic recurrence were outside the lateral margin of the external iliac vessels. No pelvic recurrence occurred around the delineations from the vessels in the direction of the pelvic cavity in the reduced margin pelvic radiotherapy group.

**Fig. 3:**
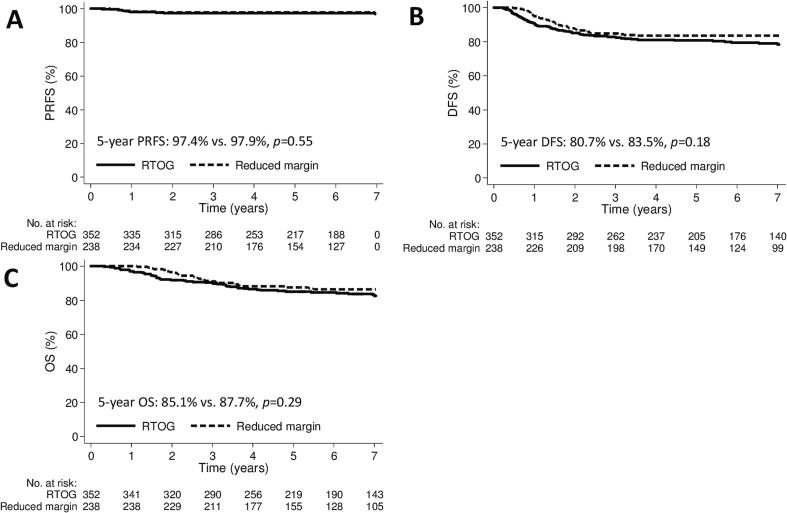
Kaplan–Meier curve demonstrating (A) PRFS, (B) DFS, and (C) OS according to the pelvic radiotherapy groups. DFS, disease-free survival; OS, overall survival; PRFS, pelvic recurrence-free survival.

The 5-year PRFS, DFS, and OS rates for RTOG versus reduced margin pelvic radiotherapy were 97.4% versus 97.9% (*p* = 0.55), 80.7% versus 83.5% (*p* = 0.18), and 85.1% versus 87.7% (*p* = 0.29), respectively (Fig. 3). In the subgroup analysis based on cancer type, PRFS, DFS, and OS did not significantly differ between the groups (Figure S1).

The results of the Cox proportional hazard regression analysis for survival outcomes are presented in Tables S3–S4. The multivariable Cox regression analyses revealed no effect of the delineation on DFS and OS when controlling for age, BMI, ECOG performance status, tumor stage, histology, types of surgery, chemotherapy use, and pelvic radiotherapy dose.

## 4 Discussion

To the best of our knowledge, this is the first study to report GI toxicity and outcomes of reduced margin pelvic radiotherapy compared with those of RTOG-based pelvic radiotherapy. Reduced margin pelvic nodal delineation significantly lowers the radiation dose to the small bowel and rectum. This study showed that reduced margin pelvic radiotherapy was independently associated with less severe physician- or patient-reported acute GI toxicities. Reduced margin pelvic radiotherapy was also associated with a lower incidence of severe late GI toxicity. The pelvic recurrence rate and survival outcomes were similar between the reduced margin and RTOG pelvic radiotherapy groups.

The RTOG-1203 and PARCER trials have established the clinical benefits of pelvic IMRT over conventional pelvic radiotherapy. The pelvic nodal clinical target volumes of these trials were delineated with a uniform 7 mm expansion surrounding the pelvic vessels. Comparing our physician-graded acute GI toxicity with those studies, the proportion of patients with any grade ≥ 2 acute GI toxicity was similar in the RTOG pelvic radiotherapy group, but lower in the reduced margin pelvic radiotherapy group [5], [6]. Furthermore, we evaluated the effect of reduced margin pelvic radiotherapy using PRO-CTCAE, because patient-reported outcomes can provide a more accurate assessment of treatment-related toxicity [15], [16], [17], [18], [19]. The proportion of patients who reported frequent diarrhea was 33.7% in the RTOG-1203 trial and 24.2% among our patients who received RTOG pelvic radiotherapy, but a lower proportion of patients received reduced margin pelvic radiotherapy (10.8%). The proportion of severe patient-reported abdominal pain (severity and interference) in patients who received RTOG pelvic radiotherapy was similar to that in the RTOG-1203 trial, whereas our patients who received reduced margin pelvic radiotherapy reported less severe abdominal pain (severity and interference).

Severe late GI toxicity can considerably affect patients’ quality of life and may require surgical intervention. Pelvic IMRT also reduces the risk of severe late GI toxicity [5], [6], [7], [8], [9]. This study showed that the proportion of patients who received RTOG pelvic radiotherapy had a similar incidence of severe late GI toxicity as that of previous studies, while the corresponding proportion of patients who received reduced margin pelvic radiotherapy was lower. These findings suggest that a decrease in the radiation dose to the bowel by reduced margin pelvic radiotherapy may translate to less acute and late GI toxicities in pelvic IMRT. Our findings should be evaluated in future studies.

The reduced margin pelvic radiotherapy had a smaller margin of target volume toward pelvic cavity than the RTOG guideline, which may raise concerns regarding the risk of pelvic recurrence. However, this study showed that RTOG and reduced margin pelvic radiotherapy resulted in similar PRFS, OS, and DFS. In the subgroup analysis based on tumor type, there were also no differences in survival outcomes between groups in patients with cervical or endometrial cancer. The treatment outcomes in this study were comparable to those of previous studies [6], [7], [8]. A possible explanation for the similar outcomes of reduced margin pelvic radiotherapy is that the anatomical distribution of lymph nodes around the pelvic vessels is non-uniform. Based on the mapping of pelvic nodes in a previous study [12], pelvic nodes are more likely to be laterally distributed and less likely to be distributed toward the direction of the pelvic cavity. Therefore, the current results support our hypothesis that reduced margin pelvic radiotherapy reduces GI toxicity without jeopardizing outcomes.

This study has some limitations. As this was a retrospective analysis of the data, selection bias may have been present. The prospective designs of inclusion of patients, power analysis, and randomization were not performed in this study. Notably, the number of patients in the two groups was not balanced because of the preference of some physicians for the RTOG guideline, suggesting that the physicians’ preference may bring confounding bias concerning survival and recurrence into this study. Although this study analyzed 590 patients who underwent hysterectomy and adjuvant pelvic radiotherapy at two tertiary centers, the current results of this study may not be able to draw firm conclusions. Further studies with a larger patient cohort and including more institutions are necessary to validate the findings of this study. The strength of this study is that GI toxicities were assessed using both physician- and patient-reported outcomes, as these can provide a more comprehensive view of treatment-related toxicity assessments. This study had an adequate follow-up period, with outcomes comparable to those of previous studies [5], [6], [7], [8], [9].

This study provides evidence that reduced margin pelvic radiotherapy decreases GI toxicity and achieves favorable outcomes in women receiving adjuvant pelvic radiotherapy for gynecological cancer. The current study used a planning target volume margin of 7 mm around pelvic nodal clinical target volume, while the EMBRACE II study had suggested a planning target volume margin of 5 mm [20]. A reduction in the margin of the planning target volume may further reduce radiation dose to the bowel and potentially lower gastrointestinal toxicity. Furthermore, the implementation of novel radiotherapy modalities such as automated treatment planning software and daily adaptation to the target volume may further reduce the radiation dose to the small bowel and GI toxicity [21]. Proton beam radiotherapy can potentially provide optimized outcomes [22].

## 5 Conclusions

This study revealed that reduced margin pelvic radiotherapy decreased the radiation dose to the bowel and this resulted in reduced acute and late GI toxicity, when compared with that of RTOG pelvic radiotherapy. The pelvic control and survival outcomes were similar between the reduced margin and RTOG pelvic radiotherapy groups in this study. Reduced margin pelvic radiotherapy may optimize treatment outcomes in women receiving adjuvant pelvic radiotherapy for gynecological cancer.

## Declaration of Competing Interest

The authors declare that they have no known competing financial interests or personal relationships that could have appeared to influence the work reported in this paper.
