# Supplementary material for: Impact of reduced margin pelvic radiotherapy on gastrointestinal toxicity and outcome in gynecological cancer
Source: Clin Transl Radiat Oncol. 2023 Aug 28;43:100671. doi: 10.1016/j.ctro.2023.100671 (PMC10482739; doi:10.1016/j.ctro.2023.100671)

**Supplementary Table 1** Logistic regression analyses for physician-reported acute GI toxicity (*n*=590).

|  | **Any CTCAE grade ≥ 2 GI toxicity** | | | | |  | **Any CTCAE grade 3 GI toxicity** | | | | |
| --- | --- | --- | --- | --- | --- | --- | --- | --- | --- | --- | --- |
|  | Univariable |  |  | Multivariable |  |  | Univariable |  |  | Multivariable |  |
|  | OR (95% CI) | *p* value |  | OR (95% CI) | *p* value |  | OR (95% CI) | *p* value |  | OR (95% CI) | *p* value |
| **Age, continuous** | 0.99 (0.98-1.02) | 0.72 |  | 1.01 (0.99-1.03) | 0.33 |  | 1.04 (1.01-1.08) | 0.02 |  | 1.05 (1.02-1.09) | 0.006 |
| **BMI, continuous** | 0.80 (0.75-0.87) | <0.001 |  | 0.78 (0.72-0.85) | <0.001 |  | 0.65 (0.56-0.76) | <0.001 |  | 0.61 (0.51-0.73) | <0.001 |
| **ECOG (0 vs. 1)** | 0.89 (0.52-1.53) | 0.67 |  | 0.97 (0.54-1.75) | 0.91 |  | 1.23 (0.50-3.06) | 0.65 |  | 1.36 (0.50-3.74) | 0.55 |
| **Tumor site (cervix vs. endometrium)** | 0.92 (0.62-1.36) | 0.66 |  | 1.69 (0.90-3.17) | 0.11 |  | 0.68 (0.34-1.36) | 0.27 |  | 1.78 (0.58-5.47) | 0.32 |
| **Types of surgery (ref: TAH/BSO + LND)** |  |  |  |  |  |  |  |  |  |  |  |
| TLH/BSO + LND | 1.06 (0.66-1.71) | 0.81 |  | 1.12 (0.68-1.86) | 0.66 |  | 0.52 (0.21-1.30) | 0.16 |  | 0.55 (0.20-1.53) | 0.25 |
| RH/BSO + LND | 1.28 (0.81-2.03) | 0.29 |  | 1.33 (0.70-2.53) | 0.39 |  | 1.05 (0.49-2.27) | 0.90 |  | 0.94 (0.29-3.07) | 0.92 |
| **Chemotherapy (no vs. yes)** | 1.38 (0.96-1.99) | 0.09 |  | 1.24 (0.83-1.84) | 0.29 |  | 0.85 (0.43-1.70) | 0.65 |  | 0.64 (0.30-1.39) | 0.26 |
| **RT dose (45Gy vs. 50.4Gy)** | 0.86 (0.55-1.35) | 0.52 |  | 0.86 (0.53-1.38) | 0.52 |  | 0.81 (0.35-1.88) | 0.62 |  | 0.88 (0.36-2.19) | 0.79 |
| **Delineation (RTOG vs. reduced margin)** | 0.39 (0.26-0.59) | <0.001 |  | 0.38 (0.25-0.58) | <0.001 |  | 0.33 (0.14-0.75) | 0.009 |  | 0.28 (0.11-0.69) | 0.005 |

Abbreviations: BMI, body mass index; BSO, bilateral salpingo-oophorectomy; CI, confidence interval; CTCAE, common terminology criteria for adverse events; ECOG, Eastern Cooperative Oncology Group; GI, gastrointestinal; OR, odds ratio; RH, radical hysterectomy; RT, radiotherapy; TAH, total abdominal hysterectomy; TLH, total laparoscopic hysterectomy.

**Supplementary Table 2** Logistic regression analyses for patient-reported acute GI toxicity (*n*=298).

|  | **Any PRO-CTCAE score 3 GI toxicity** | | | | |  | **PRO-CTCAE score 3 diarrhea** | | | | |
| --- | --- | --- | --- | --- | --- | --- | --- | --- | --- | --- | --- |
|  | Univariable |  |  | Multivariable |  |  | Univariable |  |  | Multivariable |  |
|  | OR (95% CI) | *p* value |  | OR (95% CI) | *p* value |  | OR (95% CI) | *p* value |  | OR (95% CI) | *p* value |
| **Age, continuous** | 1.02 (0.99-1.04) | 0.26 |  | 1.04 (1.01-1.08) | 0.01 |  | 1.01 (0.98-1.04) | 0.60 |  | 1.03 (0.99-1.07) | 0.06 |
| **BMI, continuous** | 0.70 (0.62-0.79) | <0.001 |  | 0.65 (0.56-0.75) | <0.001 |  | 0.66 (0.57-0.76) | <0.001 |  | 0.62 (0.53-0.73) | <0.001 |
| **ECOG (0 vs. 1)** | 0.63 (0.27-1.48) | 0.28 |  | 0.73 (0.28-1.88) | 0.51 |  | 0.51 (0.19-1.36) | 0.18 |  | 0.63 (0.22-1.84) | 0.40 |
| **Tumor site (cervix vs. endometrium)** | 0.78 (0.43-1.38) | 0.39 |  | 1.53 (0.54-4.30) | 0.42 |  | 0.68 (0.37-1.25) | 0.21 |  | 1.68 (0.56-5.04) | 0.35 |
| **Types of surgery (ref: TAH/BSO + LND)** |  |  |  |  |  |  |  |  |  |  |  |
| TLH/BSO + LND | 0.72 (0.34-1.54) | 0.40 |  | 1.04 (0.42-2.54) | 0.94 |  | 0.65 (0.29-1.48) | 0.31 |  | 0.94 (0.36-2.46) | 0.91 |
| RH/BSO + LND | 1.03 (0.48-2.20) | 0.95 |  | 0.98 (0.31-3.08) | 0.97 |  | 1.15 (0.52-2.56) | 0.73 |  | 1.11 (0.33-3.73) | 0.87 |
| **Chemotherapy (no vs. yes)** | 0.98 (0.57-1.70) | 0.95 |  | 0.80 (0.41-1.56) | 0.52 |  | 0.93 (0.52-1.67) | 0.81 |  | 0.72 (0.35-1.46) | 0.36 |
| **RT dose (45Gy vs. 50.4Gy)** | 0.86 (0.45-1.65) | 0.65 |  | 0.98 (0.48-2.01) | 0.96 |  | 0.79 (0.39-1.60) | 0.51 |  | 0.90 (0.41-1.95) | 0.79 |
| **Delineation (RTOG vs. reduced margin)** | 0.36 (0.19-0.67) | 0.001 |  | 0.29 (0.14-0.60) | 0.001 |  | 0.38 (0.19-0.75) | 0.005 |  | 0.33 (0.15-0.71) | 0.004 |

Abbreviations: BMI, body mass index; CI, confidence interval; BSO, bilateral salpingo-oophorectomy; CTCAE, common terminology criteria for adverse events; ECOG, Eastern Cooperative Oncology Group; GI, gastrointestinal; OR, odds ratio; RH, radical hysterectomy; RT, radiotherapy; TAH, total abdominal hysterectomy; TLH, total laparoscopic hysterectomy.

**Supplementary Table 3** Cox regression analyses for disease-free survival (*n*=590).

|  | Univariable |  |  | Multivariable model A* |  |  | Multivariable model B* |  |
| --- | --- | --- | --- | --- | --- | --- | --- | --- |
|  | HR (95% CI) | *p* value |  | HR (95% CI) | *p* value |  | HR (95% CI) | *p* value |
| **Age, continuous** | 1.01 (0.99-1.03) | 0.34 |  | 1.02 (0.99-1.04) | 0.12 |  | 1.01 (0.99-1.03) | 0.21 |
| **BMI, continuous** | 0.94 (0.88-1.01) | 0.10 |  | 0.96 (0.89-1.03) | 0.25 |  | 0.95 (0.88-1.02) | 0.16 |
| **ECOG (0 vs. 1)** | 1.51 (0.94-2.42) | 0.09 |  | 1.52 (0.92-2.51) | 0.10 |  | 1.57 (0.95-2.59) | 0.08 |
| **Tumor stage**  **(Reference: endometrium FIGO stage I)** |  |  |  |  |  |  |  |  |
| Endometrium, FIGO stage II | 1.12 (0.46-2.74) | 0.81 |  | 2.52 (0.89-7.20) | 0.08 |  |  |  |
| Endometrium, FIGO stage III | 2.94 (1.65-5.22) | <0.001 |  | 2.30 (0.74-7.14) | 0.15 |  |  |  |
| Cervix, FIGO stage I | 2.37 (1.27-4.42) | 0.007 |  | 3.11 (1.27-7.62) | 0.01 |  |  |  |
| Cervix, FIGO stage II | 1.55 (0.63-3.81) | 0.34 |  | 2.17 (0.93-5.07) | 0.07 |  |  |  |
| **Histology (ref: endometrioid, grade 1)** |  |  |  |  |  |  |  |  |
| Endometrioid, grade 2 | 1.84 (0.68-4.94) | 0.23 |  |  |  |  | 1.90 (0.70-5.14) | 0.21 |
| Endometrioid, grade 3 | 4.49 (1.75-11.53) | 0.002 |  |  |  |  | 4.47 (1.74-11.51) | 0.002 |
| Serous/clear cell/other | 6.79 (2.54-18.20) | <0.001 |  |  |  |  | 5.52 (2.04-14.96) | 0.001 |
| Cervical, squamous cell carcinoma | 3.11 (1.19-8.15) | 0.02 |  |  |  |  | 1.83 (0.64-5.21) | 0.26 |
| Cervical, adenocarcinoma | 4.11 (1.45-11.67) | 0.008 |  |  |  |  | 2.40 (0.78-7.39) | 0.13 |
| **Types of surgery (ref: TAH/BSO + LND)** |  |  |  |  |  |  |  |  |
| TLH/BSO + LND | 0.99 (0.58-1.71) | 0.98 |  | 0.92 (0.54-1.59) | 0.77 |  | 0.97 (0.56-1.68) | 0.92 |
| RH/BSO + LND | 1.82 (1.13-2.93) | 0.01 |  | 2.35 (1.32-4.18) | 0.004 |  | 2.04 (1.15-3.64) | 0.02 |
| **Chemotherapy (no vs. yes)** | 2.67 (1.81-3.93) | <0.001 |  | 2.45 (1.37-4.18) | 0.002 |  | 2.50 (1.68-3.72) | <0.001 |
| **RT dose (45Gy vs. 50.4Gy)** | 1.29 (0.84-1.97) | 0.24 |  | 0.81 (0.53-1.24) | 0.34 |  | 0.75 (0.49-1.15) | 0.18 |
| **Delineation (RTOG vs. reduced margin)** | 0.77 (0.52-1.14) | 0.19 |  | 0.72 (0.49-1.08) | 0.11 |  | 0.72 (0.49-1.07) | 0.11 |

Abbreviations: BMI, body mass index; BSO, bilateral salpingo-oophorectomy; CI, confidence interval; ECOG, Eastern Cooperative Oncology Group; FIGO, International Federation of Gynecology and Obstetrics; GI, gastrointestinal; HR, hazard ratio; LND, lymph node dissection; RH, radical hysterectomy; RT, radiotherapy; TAH, total abdominal hysterectomy; TLH, total laparoscopic hysterectomy.

* The tumor stage and histology were separately analyzed in multivariable models A and B, because significant interaction occurred between these two covariables.

**Supplementary Table 4** Cox regression analyses for overall survival (*n*=590).

|  | Univariable |  |  | Multivariable model A |  |  | Multivariable model B |  |
| --- | --- | --- | --- | --- | --- | --- | --- | --- |
|  | HR (95% CI) | *p* value |  | HR (95% CI) | *p* value |  | HR (95% CI) | *p* value |
| **Age, continuous** | 1.03 (1.00-1.05) | 0.03 |  | 1.03 (1.01-1.05) | 0.01 |  | 1.03 (1.00-1.05) | 0.02 |
| **BMI, continuous** | 0.94 (0.86-1.02) | 0.12 |  | 0.94 (0.87-1.03) | 0.16 |  | 0.93 (0.86-1.02) | 0.11 |
| **ECOG (0 vs. 1)** | 1.53 (0.89-2.63) | 0.13 |  | 1.46 (0.82-2.60) | 0.20 |  | 1.53 (0.86-2.71) | 0.15 |
| **Tumor stage**  **(Reference: endometrium FIGO stage I)** |  |  |  |  |  |  |  |  |
| Endometrium, FIGO stage II | 0.57 (0.19-1.72) | 0.32 |  | 0.56 (0.18-1.70) | 0.30 |  |  |  |
| Endometrium, FIGO stage III | 1.92 (1.07-3.44) | 0.03 |  | 1.13 (0.48-2.65) | 0.78 |  |  |  |
| Cervix, FIGO stage I | 1.72 (0.90-3.27) | 0.10 |  | 0.92 (0.40-2.10) | 0.84 |  |  |  |
| Cervix, FIGO stage II | 1.04 (0.38-2.83) | 0.95 |  | 0.42 (0.13-1.38) | 0.15 |  |  |  |
| **Histology (ref: endometrioid, grade 1)** |  |  |  |  |  |  |  |  |
| Endometrioid, grade 2 | 3.12 (0.92-10.59) | 0.07 |  |  |  |  | 3.05 (0.90-10.40) | 0.07 |
| Endometrioid, grade 3 | 5.27 (1.58-17.55) | 0.007 |  |  |  |  | 5.01 (1.50-16.77) | 0.009 |
| Serous/clear cell/other | 7.94 (2.26-27.86) | 0.001 |  |  |  |  | 7.21 (2.04-25.57) | 0.002 |
| Cervical, squamous cell carcinoma | 4.30 (1.27-14.54) | 0.02 |  |  |  |  | 3.01 (0.81-11.26) | 0.10 |
| Cervical, adenocarcinoma | 4.59 (1.22-17.34) | 0.02 |  |  |  |  | 3.41 (0.82-14.14) | 0.09 |
| **Types of surgery (ref: TAH/BSO + LND)** |  |  |  |  |  |  |  |  |
| TLH/BSO + LND | 0.79 (0.44-1.41) | 0.43 |  | 0.75 (0.42-1.35) | 0.34 |  | 0.80 (0.44-1.43) | 0.45 |
| RH/BSO + LND | 1.23 (0.74-2.05) | 0.43 |  | 1.47 (0.76-2.86) | 0.25 |  | 1.25 (0.65-2.41) | 0.51 |
| **Chemotherapy (no vs. yes)** | 2.00 (1.30-3.08) | 0.002 |  | 1.86 (0.95-3.65) | 0.07 |  | 1.94 (1.24-3.03) | 0.004 |
| **RT dose (45Gy vs. 50.4Gy)** | 0.83 (0.46-1.49) | 0.53 |  | 0.78 (0.48-1.27) | 0.32 |  | 0.74 (0.45-1.21) | 0.23 |
| **Delineation (RTOG vs. reduced margin)** | 0.79 (0.50-1.23) | 0.29 |  | 0.75 (0.48-1.17) | 0.20 |  | 0.74 (0.47-1.16) | 0.19 |

Abbreviations: BMI, body mass index; BSO, bilateral salpingo-oophorectomy; CI, confidence interval; ECOG, Eastern Cooperative Oncology Group; FIGO, International Federation of Gynecology and Obstetrics; GI, gastrointestinal; HR, hazard ratio; LND, lymph node dissection; RH, radical hysterectomy; RT, radiotherapy; TAH, total abdominal hysterectomy; TLH, total laparoscopic hysterectomy.

* The tumor stage and histology were separately analyzed in multivariable models A and B, because significant interaction occurred between these two covariables.

**Supplementary Figure S1** Kaplan-Meier curve demonstrating pelvic recurrence-free survival, disease-free survival, and overall survival according to the tumor types. DFS, disease-free survival; OS, overall survival; PRFS, pelvic recurrence-free survival.


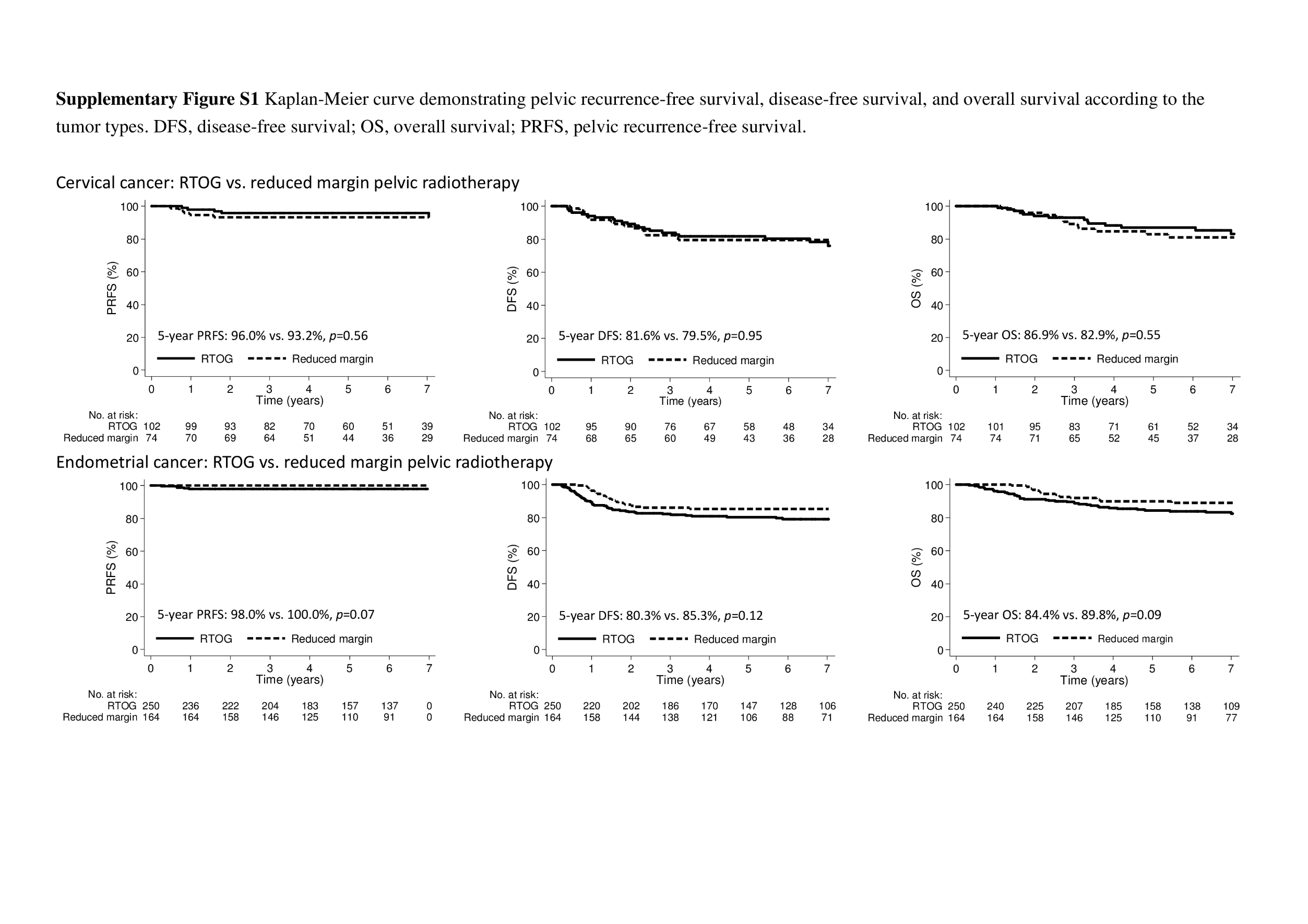

Supplement: Supplementary data 1 [file mmc1.docx]
